# Adolescents and parents experiencing parental cancer: construction of a nursing intervention model

**DOI:** 10.1590/1980-220X-REEUSP-2024-0184en

**Published:** 2025-01-13

**Authors:** Ana Filipa Domingues Sousa, Paula Cristina Moreira Mesquita de Sousa, Maria Margarida da Silva Reis dos Santos Ferreira, Maria de Lurdes Lopes de Freitas Lomba

**Affiliations:** 1 Universidade do Porto, Instituto de Ciências Biomédicas Abel Salazar, Porto, Portugal.; 2 Instituto Português de Oncologia de Coimbra, Coimbra, Portugal.; 3 Escola Superior de Enfermagem de Coimbra, Unidade de Investigação em Ciências da Saúde: Enfermagem, Coimbra, Portugal.; 4 Escola Superior de Enfermagem do Porto, Porto, Portugal.; 5 Centro de Investigação em Tecnologias e Serviços de Saúde, Porto, Portugal.

**Keywords:** Adolescent, Parents, Chancre, Nursing, Health Programs and Plans, Adolescente, Padres, Chancro, Enfermería, Planes y Programas de Salud

## Abstract

**Objective:**

To develop a nursing intervention model for adolescents and parents experiencing parental cancer.

**Method:**

Multimethod research, which integrated five studies: a scoping review and four qualitative studies. Considering the results of these studies, a nursing intervention model was constructed based on two theoretical frameworks, such as the A Model of Children’s Adjustment to Parental Cancer, for adolescents, and Neuman Systems Model, for parents, and on a semantic framework, such as Nursing Ontology.

**Results:**

The model is aimed at three target audiences: individual – parental figure with cancer; individual – adolescent; and family – parental figure with cancer, second parental figure and adolescent. The model is based on health literacy and includes four topics that address central topics in the experience of parental cancer: “The elephant in the room”; “Everything changes in us”; “Life goes on”; and “With the present, we plan the future”.

**Conclusion:**

The proposed model is assumed as a health promotion strategy that empowers nurses for a family-centered intervention, in order to minimize the impact of parental cancer.

## RESUMEN

**Objetivo:**

Desarrollar un modelo de intervención de enfermería para adolescentes y padres que experimentan cáncer parental.

**Método:**

Investigación multimétodo, que integró cinco estudios: una revisión de alcance y cuatro estudios cualitativos. Teniendo en cuenta los resultados de estos estudios, se construyó un modelo de intervención de enfermería basado en dos referentes teóricos, como son el Modelo Teórico de Adaptación Adolescente al Cáncer Parental, para adolescentes, y el Modelo de Sistemas de Neuman, para padres, y en un referente semántico, como la Ontología de Enfermería.

**Resultados:**

El modelo está dirigido a tres públicos objetivos: individuo – figura paterna con cáncer; individuo – adolescente; y familia – figura paterna con cáncer, segunda figura paterna y adolescente. El modelo se basa en la alfabetización en salud e integra cuatro temas que abordan temas centrales en la experiencia del cáncer parental: “El elefante en la habitación”; “Todo cambia en nosotros”; “La vida continua”; y “Con el presente planificamos el futuro”.

**Conclusión:**

El modelo propuesto se asume como una estrategia de promoción de la salud que capacita a enfermeros para la intervención centrada en la familia, con el fin de minimizar el impacto del cáncer parental.

## INTRODUCTION

The incidence of cancer in people under the age of 50 has increased considerably worldwide^(1)^. At this age, a cancer diagnosis has different implications than in older adults, because many are likely to still be working and have children under the age of 18^(2)^.

The experience of cancer in people with dependent children is called parental cancer (PC), a condition that has shown significant growth^(2)^. PC is a distressing life experience for the family, representing a significant stress factor for patients and children^(3,4,5,6)^. Given the physical and psychological repercussions of cancer and the inherent uncertainty about the future and potential threat to life^(7)^, PC causes suffering, changes in routines and family interaction, role reversal and socioeconomic difficulties^(3,6)^.

Adults who are struggling with PC face additional challenges throughout the disease, balancing the need to protect their children with the difficulties of continuing to fulfill their parental role. The physical and emotional toll of cancer reduces parental abilities, affecting family dynamics and making it difficult to support and communicate adequately with children^(3,8,9)^. Failure to understand children’s reactions to the diagnosis and experience of PC can also cause parents to feel like they have failed to fulfill their parental responsibilities^(6)^.

During the disease process, parental care for their children is compromised, as children and adolescents who experience PC may present more emotional and behavioral problems compared to those who do not experience this reality^(6)^. Due to their greater capacity to understand their parents’ illness, adolescents are considered more vulnerable to the experience of PC; however, their needs are not always met by a sick parent^(7,10)^. This fact can negatively affect adolescents’ psychosocial adjustment capacity, causing changes in school performance, decreasing quality of life and satisfaction, and may even lead to post-traumatic stress^(2,3)^.

Due to the burden of cancer, parents have more difficulty in monitoring and identifying atypical behaviors in adolescents, expecting health professionals to support their children in coping with PC^(3,11)^. However, in adult oncology institutions, health and child development professionals’ inexperience, the lack of knowledge and the exclusion of adolescents from the parental disease process are barriers to supporting these families^(3,11)^.

Despite the significance of this phenomenon, given the increase in the number of cases of PC and its impact on the dyad, there are no guidelines for nurses regarding intervention in adolescents and parents who experience this reality, with some authors mentioning the need to implement interventions in these families^(2,3,6,12)^.

Although scientific evidence points to barriers and difficulties for nurses in dealing with parents who experience PC and communicating with their children^(13,14)^, ignoring implications of cancer on the parental role and children’s needs can compromise the dyad’s adjustment to the new condition and the healthy development of adolescents^(14)^.

Existing intervention programs focus on parents (cancer patients), children/adolescents or the parent-child dyad (family). The results of their application in children/adolescents demonstrate the existence of significant improvements in symptoms of post-traumatic stress and depression^(11)^, quality of life, development of coping mechanisms and improvement of communication and relationship between parents and children, allowing to increase the family’s ability to deal with cancer^(4)^. The scarce nursing interventions (NIs) described in existing studies and without assessment of their effectiveness prove the need to build an intervention model that represents nursing knowledge that organizes and expresses adolescents’ and parents’ needs who experience PC.

This study aimed to develop a NI model for adolescents and parents experiencing PC.

## METHOD

A multi-method study was conducted. The first, a scoping review^(15)^, concluded that there are some intervention programs aimed at the dyad; however, they are not designed by nurses, nor are they carried out in a hospital setting. The second study^(16)^, with a qualitative approach, found that the A Model of Children’s Adjustment to Parental Cancer (AMCAPC)^(4)^ is suitable for systematizing nursing care for adolescents. The third study^(17)^, also qualitative, allowed us to determine that the Neuman Systems Model (NSM)^(18,19)^ is relevant in the approach of parents who experience PC. The fourth study, based on the qualitative paradigm^(20)^, revealed that the adolescents’ needs who have experienced PC are emotional, educational and psychosocial. The fifth qualitative study^(21)^ found that parents assumed that the experience of cancer impacted the performance of their parental role. Primary studies were carried out in an oncology hospital in the Central region of Portugal, and were approved by the Research Ethics Committee of the institution where the study was developed (Process TI 25/2020). Based on the results of these studies, the NI model for adolescents and parents experiencing PC was designed.

### Nursing Intervention Model Theoretical Frameworks and Objectives

A NI model is a conceptual framework that allows nurses to describe their practice in a given area with the purpose of focusing care on their clients.

The existence of a theoretical model that aims to systematically analyze the process of dealing with PC has implications for clinical practice^(4)^, reinforcing its relevance in identifying adolescents’ and parents’ needs based on a theoretical framework developed by nurses. Mastering nursing knowledge supported by theoretical frameworks allows for designing NIs aimed at families experiencing PC.

The NI model for adolescents and parents who experience PC is based on two theoretical frameworks: AMCAPC, for adolescents, and NSM, for parents.

According to AMCAPC, PC diagnosis causes psychological and social stress in adolescents, and the factors that influence their adjustment can be classified as moderators and mediators. The “moderator” variables refer to preexisting variables and affect the situation that generates stress, and the “mediator” variables are those that exert their influence after diagnosing the parental disease^(4)^. NIs are based on all or some of the mediating variables. The variables are influenced by the intervention, and the outcome is the response to the interaction of the moderator, mediator and NI variables. As a result, adolescents may adjust well or poorly^(4)^. NIs that consider the impact of moderating and mediating variables, or both, promote adjustment.

NIs include three items: education (about PC); normalization (creating a safe environment for expressing feelings, providing psychological support); and development of strengths (helping to recognize the ability to deal with stressful situations, facilitating the development of coping mechanisms)^(4)^.

According to NSM, an individual is an open and dynamic system, influenced by a variety of physiological, psychological, sociocultural, spiritual and developmental factors, which constantly interact with the environment^(18,19)^. The application of this model aims to assist parents in preserving, achieving and maintaining the stability of their systems. Nursing care is centered on the client system: cancer patient and family (additional client systems). As recommended by the NSM, nurses identify parents’ needs and the stimuli that may cause stress in the dyad, considering intrapersonal, interpersonal and extrapersonal factors. Based on this assessment, primary, secondary and tertiary prevention interventions are planned to maintain parents’ flexible line of defense, and nurses should subsequently assess the results of the implemented interventions^(18,19)^.

At the level of primary prevention, interventions incorporate providing information about the pathology and developing coping strategies, reducing risk factors and promoting adjustment to PC and strengthening and protecting the flexible line of defense.

Regarding secondary prevention, interventions include assessing information needs, adjustment difficulties and changes in parental role performance, enabling early diagnosis. These interventions aim to reduce reactions to stressors through resistance lines, mobilizing internal and external resources to maintain energy and achieve stability.

In the context of tertiary prevention, interventions aim to readjust to the situation, taking into account the physical and psychological limitations caused by cancer, minimizing the consequences. These interventions begin after reconstitution, allowing readjustment through communication, promotion of effective parental role performance and family coping.

This model focuses on defining NIs aimed at clients experiencing PC, seeking to guide clinical decision-making, with an emphasis on modifying meanings and improving clients’ awareness of cancer and adjusting to repercussions on family dynamics, improving family communication and providing availability and emotional support.

Considering that a NI model aims to provide a basis for the practice of client-centered care, the objectives of this model were formulated, which demonstrate the results that are intended to be achieved in the experience of PC by the family. Chart 1 describes the objectives.

**Chart 1:** Nursing intervention model objectives – Coimbra, Portugal, 2024.

Objectives of a nursing intervention model for parental cancer
Support the family experiencing parental cancer during diagnosis, treatment and follow-up.
Facilitate the health-illness transition process of a parent with cancer.
Facilitate the developmental transition process of adolescents.
Facilitate family communication and interaction.
Minimize the impact of the parental cancer experience on the family.
Facilitate the family’s adjustment to the repercussions resulting from parental cancer.

Source: authors, 2024.

The model aimed to obtain health gains for adolescents and parents, promoting their adjustment to health changes of the parental figure with cancer and in family dynamics.

The model structure includes NI organization for families experiencing PC. Considering the results of the scoping review^(15)^, the interventions outlined are based on the psychoeducational typology, which integrates the psychological, emotional and educational components, aiming to: i) increase the health literacy of parents with cancer and adolescents about PC; ii) improve the understanding of adolescents and parents with cancer about the disease; iii) minimize the family’s distress and fears related to PC; iv) promote the expression of family emotions/experiences; v) increase parental skills in communicating with adolescents; vi) guide the process of changing social roles and family dynamics.

This model is expected to be implemented by specialist nurses in the fields of child and pediatric health nursing, and mental health and psychiatric nursing.

### Methodology for Constructing the Nursing Intervention Model

The developed model aims to respond to specific objectives, consisting of a set of objectives, nursing diagnoses (ND), and autonomous NIs, built based on Nursing Ontology.

Nursing Ontology is a reference model that represents a set of concepts from the nursing disciplinary domain, as well as the relationships between them, based on relevant scientific evidence. A Nursing Ontology represents an information structure useful for the conceptual and theoretical representation of nursing knowledge. The developed Nursing Ontology can promote nursing knowledge, emphasize the need for research, allow the development of systems that facilitate the design of care and produce reliable and quality indicators associated with nursing care, reducing the time for records^(22)^. The Portuguese Order of Nurses (ON)^(23)^ also emphasizes that ontology is a significant milestone for documenting the design of care, contributing to nurses becoming increasingly significant for the population, dignifying nursing.

The model under development aims to represent nursing knowledge in PC. The model configures the significant aspects for satisfying client needs, through the representation of concepts in data, diagnoses, objectives and interventions, in an area that has been demonstrated by evidence, resulting from the developed research, representative of clients’ effective needs, in which nurses can intervene. Thus, according to the nomenclature adopted by Nursing Ontology, the model integrates three target audiences: individual (parental figure with cancer); individual (adolescent); and family (parental figure with cancer, second parental figure and adolescent).

### Nursing Intervention Model Contents

Assuming the philosophy of Family-Centered Care, which advocates individualized plans adjusted to each context, the model’s sessions range from individual (with parent with cancer or with adolescent) to group sessions, with the dyad and second parental figure.

The model consists of four topics that aim to address central topics in the different phases of the PC experience, from diagnosis, treatments and follow-up, aimed at the target audiences. Topics one and two will be addressed in individual sessions with sick parents and adolescents, and topics three and four will be addressed in group sessions with the family. Chart 2 summarizes the topics and types of sessions that make up the intervention model.

**Chart 2:** Nursing intervention model topics and session type – Coimbra, Portugal, 2024.

Topics of the nursing intervention model in parental cancer	Session type
1 - “The elephant in the room”: let’s talk about parental cancer	Individual
2 - “Everything changes in us”: how are you experiencing parental cancer?	Individual
3 - “Life goes on”: strategies for dealing with parental cancer	Group
4 - “With the present, we plan for the future”: strategies for dealing with the consequences of parental cancer throughout the course of the disease and in the future	Group

Source: authors, 2024.

The model’s design is based on the principles of health literacy, defined by the World Health Organization, as it allows clients to develop cognitive and social skills and the ability to access, understand and use information to promote and maintain health^(24)^.

Focusing on nurses’ decision-making process, the model advocates a family-centered approach based on scientific evidence. Through a partnership relationship, it is essential that nurses demonstrate knowledge, skills and abilities that promote the trust of adolescents and parents in professionals. Therefore, nurses must communicate clearly and honestly in a way that is adjusted to clients and conveys the information in an understandable manner, demonstrating respect for clients’ individuality. To this end, it is important to provide emotional support and listen actively, establishing an empathetic and cooperative relationship capable of generating trust, tranquility and a willingness of parents and adolescents to participate and improve the PC experience.

The environment in which consultations will take place must facilitate the process, and it is expected that they will take place in a suitable physical space, in which the privacy of a parent with cancer, adolescent and family is guaranteed, interruptions are avoided and it is welcoming, in line with the premise of atraumatic care.

The model is based on an active methodology to promote healthy interactions and relationships within the dyad and, at the same time, allow the acquisition of knowledge through the development of a critical and reflective spirit about reality, helping them to overcome the problems experienced.

During nursing consultations, positive reinforcement and assessment of the results of the interventions implemented are essential, allowing for improvements in the quality of care provided and reassessing the family’s needs for subsequent consultations. These strategies are important for families to value NI as a personalized care response to their problems.

## RESULTS AND DISCUSSION

The NI model for adolescents and parents experiencing PC was developed using Nursing Ontology, in line with the programs identified in the literature and the respective typology of interventions^(15)^, with the suitability of the theoretical frameworks selected for adolescents^(16)^ and parents^(17)^, with the needs described by adolescents^(20)^ and with parents’ perception of the impact of cancer on the performance of their parental role^(21)^. According to Nursing Ontology, and to respond to the needs of the dyad experiencing PC, the model comprises four stages of care design that integrate nurses’ decision-making process: data to be assessed, ND, objectives, NIs, differentiating itself at the level of three target audiences: i) Individual – parental figure with cancer; ii) Individual – adolescent; iii) Family – parental figure with cancer, second parental figure and adolescent.

### Client: Individual (Parental Figure with Cancer)

A parent with cancer experiences repercussions of the disease at various levels, namely in the body process, psychological process and action process, which can compromise the performance of their parental role, having implications for interactive behavior with their children. Therefore, nurses must implement interventions that facilitate the adjustment of parents with cancer to this experience and that promote interaction between the dyad.

In relation to the body process, it is known that cancer and its treatments have physical consequences on different organic systems, which may vary depending on the person, the type of cancer and the treatment instituted. Hence, in the design of this model, data related to the body process were not included, as what is intended to be highlighted is the impact of cancer on the performance of their parental role.

The conceptual aggregates “psychological process - emotion” and “action - interactive behavior” were identified, which resulted in data, objectives, ND and NI, aimed at facilitating the performance of the parental role during the PC experience.

### Client: Individual (Adolescent)

PC has consequences for adolescents’ life path, particularly at the level of the psychological process and the developmental transition process. Nurses must implement interventions that facilitate adolescents’ adjustive process to the PC experience. The conceptual aggregates identified were the “psychological process – emotion” and the “transition – human development – psychomotor development”, which resulted in the data, objectives, ND and NI, aimed at facilitating the experience of the critical event of PC.

### Client: Family (Parent with Cancer, Second Parent and Adolescent)

The experience of PC implies changes in the family process, in which nurses must implement interventions aimed at the parental figure with cancer, second parental figure and adolescent, to promote and facilitate the adjustive process to the disease situation.

In Nursing Ontology, interventions aimed at families experiencing a chronic illness of one of their members are not foreseen. With the construction of an intervention model and the study carried out within the scope of PC, this can constitute an impulse for the integration of a new conceptual aggregate in the family process “Preparing the family to adjust to the repercussions of cancer of a family member”, identifying the respective data, objectives, ND and NI. This proposal was supported by the results of the scoping review^(15)^ and qualitative studies^(16,17,20,21)^, which, by showing that PC impacts the entire family, intervention programs should not focus exclusively on parents and/or children, but rather be directed at the family nucleus. Thus, it was considered pertinent that this intervention model also be directed at the family, and not exclusively at the parental figure with cancer and the adolescent child, allowing the inclusion of a second parental figure.

**Figure 1:**
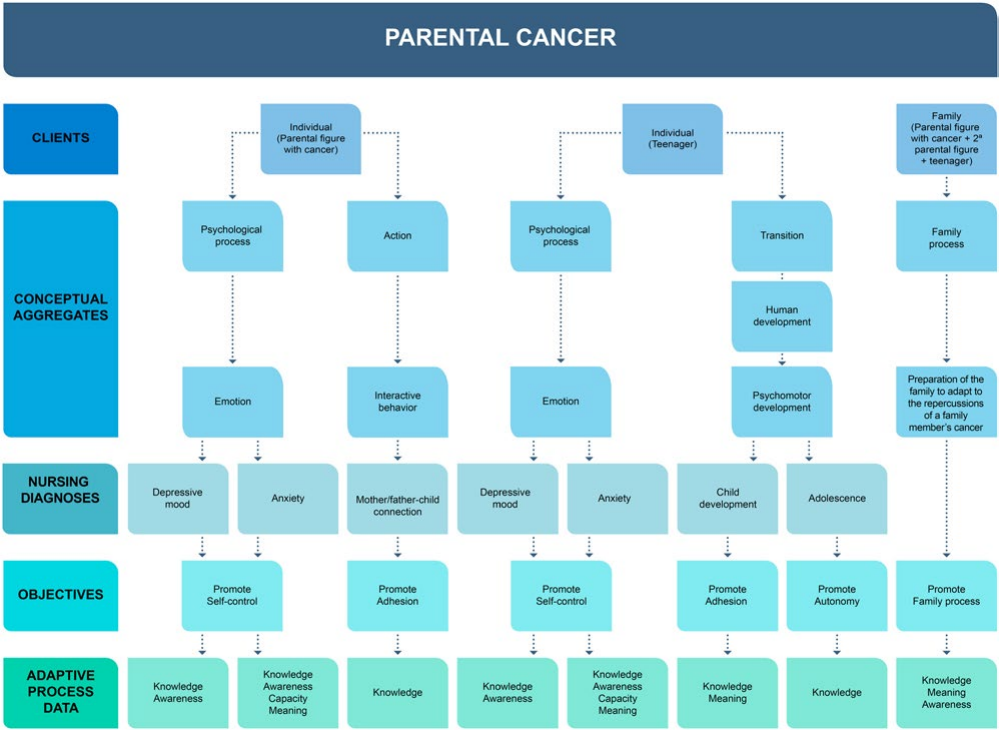
Nursing intervention model for adolescents and parents experiencing parental cancer. Coimbra, Portugal, 2024.

Figure 1 schematically illustrates the NI model that had Nursing Ontology as its conceptual, theoretical and semantic framework, representing significant nursing knowledge for adolescents and parents who experience PC.

The sequence of sessions carried out by nurses will be determined according to the initial assessment, with a first session being held with parents with cancer, a second with adolescents, and a third with the family. The subsequent sessions will depend on the assessment carried out in the first sessions, and there is no pre-set number of sessions. They may take place in the consultation service, Day Hospital and/or medical/surgical hospitalization, and may be provided either during the diagnosis and/or treatment phase of parents.

NI requires a collaborative approach between adolescents and their family, with nurses taking over as facilitator of learning and vehicle of knowledge transmission, teaching, training and empowering the dyad. In clinical practice, the implementation of projects stands out as a contribution to good practices, in which nurses can apply their knowledge, assuming that, in a given context, some solutions may contribute, compared to others, to the resolution of potential problems^(25)^.

Given the results of the scoping review^(15)^, it was considered pertinent that the intervention model also targeted the family, and not exclusively the parental figure with cancer and adolescent child, enabling the inclusion of a second parental figure.

It is important to highlight that Nursing Ontology integrates the ICNP^®^ taxonomy^(26)^ for mapping the concepts of statements, and the ND and NI foci, identified in studies carried out with an adolescent^(16)^ and parent with cancer^(17)^ experiencing PC, were considered in the model design.

The model development was based on psychoeducational interventions, because, in accordance with the results of the scoping review^(15)^, this type of intervention is not only the most predominant, but also appears to best respond to the needs of the dyad facing PC.

Concerning the conceptual aggregates identified for the different clients, the aim was to meet the objectives related to the condition and outcome objectives of the adjustive process to minimize the effects resulting from the experience of PC through knowledge, meaning, capacity and awareness. The NIs outlined aim to respond to adolescents’ educational, emotional and psychosocial needs^(20)^ and facilitate the performance of the parental role of parents with cancer^(21)^. From the development of this model, the proposal resulted to integrate a new conceptual aggregate into the family process: “Preparing the family to adjust to the repercussions of a family member’s cancer”. This aggregate, which can be integrated into Nursing Ontology, is relevant given the exponential growth of PC and the fact that NI is significant for clients who experience this phenomenon.

The limitations of this study are related to its geographical limitation and the fact that the studies that allowed us to assess the suitability of AMCAPC for adolescents and the relevance of NSM in the approach to parents were carried out with a single participant. It is also worth noting the fact that the model has not yet been implemented, considering another limitation of this study.

It is suggested that further research be carried out in different populations, characterizing them and identifying their specific needs in advance in order to direct interventions to the problems assessed. It is also recommended that other studies explore the levels of intervention of each health professional in this model.

Knowledge of the phenomenon and its current relevance should encourage the implementation of this model for families experiencing PC in hospitals, where the parental figure with cancer is monitored.

## CONCLUSION

This study made it possible to develop a NI model for adolescents and parents who experience PC based on scientific evidence resulting from studies carried out, which highlighted the needs of adolescents who experience PC and the impact of parents’ illness on the performance of their parental role. The model is supported by theoretical nursing frameworks and its conception, considering Nursing Ontology as a conceptual and semantic framework.

Nurses’ professional practice is part of a vast context of intervention, and nurses must take on an innovative and entrepreneurial role. Thus, as an implication for clinical practice, the creation of this intervention model emerges as a health-promoting strategy that enables nurses to perform family-centered interventions. In this context, it is believed that the results of this research can be used to reformulate health and education policies based on NI implementation in different contexts, minimizing the consequences of PC on the dyad and family that experiences it.
