# Supplementary material for: Adolescents and parents experiencing parental cancer: construction of a nursing intervention model
Source: Rev Esc Enferm USP. 2025 Jan 13;58:e20240184. doi: 10.1590/1980-220X-REEUSP-2024-0184en (PMC11774517; doi:10.1590/1980-220X-REEUSP-2024-0184en)
Supplement: Translation

# Translation: Adolescentes e pais que vivenciam cancro parental: construção de um modelo de intervenção de enfermagem

**DOI:** 10.1590/1980-220X-REEUSP-2024-0184pt

**Authors:** Ana Filipa Domingues Sousa, Paula Cristina Moreira Mesquita de Sousa, Maria Margarida da Silva Reis dos Santos Ferreira, Maria de Lurdes Lopes de Freitas Lomba

**Affiliations:** 1 Universidade do Porto, Instituto de Ciências Biomédicas Abel Salazar, Porto, Portugal.; 2 Instituto Português de Oncologia de Coimbra, Coimbra, Portugal.; 3 Escola Superior de Enfermagem de Coimbra, Unidade de Investigação em Ciências da Saúde: Enfermagem, Coimbra, Portugal.; 4 Escola Superior de Enfermagem do Porto, Porto, Portugal.; 5 Centro de Investigação em Tecnologias e Serviços de Saúde, Porto, Portugal.

**Keywords:** Adolescente, Pais, Cancro, Enfermagem, Planos e Programas de Saúde

## Abstract

**Objetivo:**

Desenvolver modelo de intervenção de enfermagem para adolescentes e pais que vivenciam cancro parental.

**Método:**

Investigação multimétodos, que integrou cinco estudos: uma *scoping review* e quatro estudos qualitativos. Atendendo aos resultados destes estudos, construiu-se um modelo de intervenção de enfermagem sustentado em dois referenciais teóricos, como Modelo Teórico do Ajuste dos Adolescentes ao Cancro Parental, para os adolescentes, e Modelo de Sistemas de Neuman, para os pais, e em um referencial semântico, como a Ontologia de Enfermagem.

**Resultados:**

O modelo dirige-se a três públicos-alvo: indivíduo – figura parental com cancro; indivíduo – adolescente; e família – figura parental com cancro, segunda figura parental e adolescente. O modelo baseia-se na literacia em saúde e integra quatro tópicos que abordam temas centrais na vivência do cancro parental: “O elefante na sala”; “Tudo muda em nós”; “A vida continua”; e “Com o presente, planejamos o futuro”.

**Conclusão:**

O modelo proposto assume-se como uma estratégia de promoção da saúde que capacita os enfermeiros para uma intervenção centrada na família, de forma a minimizar o impacto do cancro parental.

## INTRODUÇÃO

A incidência de cancro em pessoas com idade inferior a 50 anos aumentou consideravelmente em todo o mundo^(1)^. Nessas idades, um diagnóstico de cancro tem implicações distintas relativamente às encontradas nas pessoas mais velhas, porque provavelmente muitas ainda trabalham e são pais de filhos menores de 18 anos^(2)^.

A vivência de cancro nas pessoas com filhos dependentes é denominada de cancro parental (CP), uma condição que tem apresentado um crescimento significativo^(2)^. O CP é uma experiência de vida angustiante para a família, representando um fator de estresse significativo para pacientes e filhos^(3,4,5,6)^. Diante das repercussões físicas e psicológicas do cancro e da inerente incerteza sobre o futuro e potencial de ameaça à vida^(7)^, o CP causa sofrimento, alterações nas rotinas e na interação familiar, inversão de papéis e dificuldades socioeconômicas^(3,6)^.

Os adultos que enfrentam o CP deparam-se com desafios adicionais ao longo da doença, equilibrando a necessidade de proteger os seus filhos com as dificuldades em continuar a desempenhar o papel parental. O desgaste físico e emocional provocado pelo cancro reduz as capacidades parentais, afetando a dinâmica familiar e dificultando o apoio e a comunicação adequada com os filhos^(3,8,9)^. A incompreensão das reações dos filhos ao diagnóstico e vivência do CP pode também causar nos pais sentimentos de fracasso na assunção das suas responsabilidades parentais^(6)^.

No processo de doença, o cuidado parental aos seus filhos fica comprometido, pois as crianças e adolescentes que se deparam com o CP podem apresentar mais problemas emocionais e comportamentais comparativamente às que não experienciam esta realidade^(6)^. Devido à sua maior capacidade de compreensão sobre a doença dos pais, os adolescentes são considerados mais vulneráveis à experiência do CP, no entanto nem sempre as suas necessidades são atendidas pelo progenitor doente^(7,10)^. Este fato pode afetar negativamente a capacidade de ajuste psicossocial dos adolescentes, provocando alterações no desempenho escolar, diminuindo a qualidade e satisfação com a vida, podendo, inclusive, originar estresse pós-traumático^(2,3)^.

Pela sobrecarga da doença oncológica, os pais têm mais dificuldade em acompanhar e identificar comportamentos atípicos nos adolescentes, esperando que os profissionais de saúde apoiem os seus filhos no enfrentamento do CP^(3,11)^. Contudo, nas instituições oncológicas de adultos, a inexperiência dos profissionais de saúde e desenvolvimento infantil, o déficit no conhecimento e a exclusão dos adolescentes do processo de doença parental são barreiras ao apoio a essas famílias^(3,11)^.

Não obstante a significância deste fenômeno, diante do acréscimo do número de casos de CP e do seu impacto na díade, não existem orientações para os enfermeiros referentes à intervenção nos adolescentes e pais que experienciam esta realidade, mencionando alguns autores a necessidade de implementar intervenções nessas famílias^(2,3,6,12)^.

Apesar de a evidência científica apontar barreiras e dificuldades dos enfermeiros em lidar com os pais que vivenciam o CP e de comunicar com os seus filhos^(13,14)^, ignorar as implicações do cancro no papel parental e as necessidades dos filhos pode comprometer a adaptação da díade à nova condição e o desenvolvimento saudável dos adolescentes^(14)^.

Os programas de intervenção existentes centram-se nos pais (doentes oncológicos), crianças/adolescentes ou na díade pais e filhos (família). Os resultados da sua aplicação nas crianças/adolescentes demonstram a existência de melhorias significativas nos sintomas de estresse pós-traumático e depressão^(11)^, qualidade de vida, desenvolvimento de mecanismos de *coping* e aperfeiçoamento da comunicação e relacionamento entre pais e filhos, permitindo aumentar a capacidade da família para lidar com o cancro^(4)^. As parcas intervenções de enfermagem (IEs) descritas nos estudos existentes e sem avaliação da sua efetividade comprovam a necessidade de construir um modelo de intervenção que represente o conhecimento de enfermagem que organiza e expressa as necessidades dos adolescentes e pais que vivenciam o CP.

O objetivo deste estudo foi desenvolver um modelo de IE para adolescentes e pais que vivenciam o CP.

## MÉTODO

Realizou-se estudo multimétodos. O primeiro, uma *scoping review* ^(15)^, concluiu que existem alguns programas de intervenção dirigidos à díade, contudo não são desenhados por enfermeiros, nem se realizam em ambiente hospitalar. No segundo estudo^(16)^, de abordagem qualitativa, constatou-se que o Modelo Teórico do Ajuste dos Adolescentes ao Cancro Parental (MTAACP)^(4)^ é adequado para sistematizar os cuidados de enfermagem aos adolescentes. O terceiro estudo^(17)^, também qualitativo, permitiu aferir que o Modelo de Sistemas de Neuman (MSN)^(18,19)^ é pertinente na abordagem dos pais que experienciam o CP. O quarto estudo, assente no paradigma qualitativo^(20)^, revelou que as necessidades que os adolescentes que vivenciaram o CP apresentam são do tipo emocional, educacional e psicossocial. No quinto estudo qualitativo^(21)^, verificou-se que os pais assumiam que a vivência do cancro impactava o desempenho do seu papel parental. Os estudos primários foram realizados em hospital oncológico da região do Centro de Portugal, e foram aprovados pela Comissão de Ética em Pesquisa da instituição onde se desenvolveu o estudo (Processo n^o^ TI 25/2020). Com base nos resultados desses estudos, desenhou-se o modelo de IE para adolescentes e pais que vivenciam o CP.

### Referenciais Teóricos e Objetivos do Modelo de Intervenção de Enfermagem

Um modelo de IE é uma base conceptual que permite aos enfermeiros descrever a sua prática em determinada área com o propósito de centrar os cuidados aos seus clientes.

A existência de um modelo teórico que objetive analisar sistematicamente o processo de lidar com o CP apresenta implicações na prática clínica^(4)^, reforçando a sua relevância na identificação das necessidades dos adolescentes e pais com base em referencial teórico desenvolvido por enfermeiros. O domínio do conhecimento da disciplina de enfermagem sustentado em referenciais teóricos permite delinear IEs dirigidas às famílias que vivenciam o CP.

O modelo de IE para adolescentes e pais que vivenciam o CP é sustentado em dois referenciais teóricos: o MTAACP, para os adolescentes, e o MSN, para os pais.

Segundo o MTAACP, o diagnóstico de CP origina estresse psicológico e social nos adolescentes, e os fatores que influenciam a sua adaptação podem ser classificados como moderadores e mediadores. As variáveis “moderadoras” reportam-se às preexistentes e afetam a situação geradora de estresse, e as variáveis “mediadoras” são as que exercem a sua influência após o diagnóstico da doença parental^(4)^. As IEs têm como base todas ou algumas das variáveis mediadoras. As variáveis são influenciadas pela intervenção, e o resultado é a resposta à interação das variáveis moderadoras, mediadoras e IEs. Como resultado, pode verificar-se um bom ou mau ajuste dos adolescentes^(4)^. As IEs que considerem o impacto de variáveis moderadoras e mediadoras, ou ambas, promovem o ajuste.

As IEs integram três itens: educação (sobre o CP); normalização (criação de um ambiente seguro para a expressão de sentimentos, disponibilizando apoio psicológico); e desenvolvimento dos pontos fortes (auxiliando a reconhecer a capacidade de lidar com situações de estresse, facilitando o desenvolvimento de mecanismos de *coping*)^(4)^.

De acordo com o MSN, o indivíduo é um sistema aberto e dinâmico, influenciado por uma variedade de fatores fisiológicos, psicológicos, socioculturais, espirituais e desenvolvimentais, que interagem constantemente com o ambiente^(18,19)^. Com a aplicação deste modelo, pretende-se auxiliar os pais na preservação, alcance e manutenção da estabilidade dos seus sistemas. Os cuidados de enfermagem são centrados no sistema cliente: doente oncológico e família (sistemas de clientes adicionais). Conforme preconizado pelo MSN, o enfermeiro identifica as necessidades dos pais e os estímulos que possam causar estresse na díade, considerando fatores intrapessoais, interpessoais e extrapessoais. Com base nessa avaliação, são planejadas intervenções de prevenção primária, secundária e terciária para manter a linha flexível de defesa dos pais, devendo o enfermeiro, posteriormente, avaliar o resultado das intervenções implementadas^(18,19)^.

Ao nível da prevenção primária, as intervenções incorporam prover informação sobre a patologia e desenvolvimento de estratégias de *coping*, reduzindo fatores de risco e promovendo a adaptação ao CP e pelo fortalecimento e proteção da linha flexível de defesa.

Relativamente à prevenção secundária, as intervenções contemplam a avaliação das necessidades de informação, dificuldades de adaptação e alterações no desempenho do papel parental, permitindo um diagnóstico precoce. Estas intervenções tencionam reduzir a reação aos estressores, através das linhas de resistência, mobilizando recursos internos e externos para manter a energia e atingir a estabilidade.

No âmbito da prevenção terciária, as intervenções objetivam a readaptação à situação, atendendo às limitações físicas e psicológicas causadas pelo cancro, minimizando as consequências. Estas intervenções iniciam-se após a reconstituição, permitindo a readaptação, através da comunicação, promoção do desempenho de papel parental efetivo e *coping* familiar.

Este modelo focaliza-se na definição das IE dirigidas para clientes vivenciando o CP, procurando guiar a tomada de decisão clínica, com ênfase na modificação de significados e melhoria da consciencialização dos clientes sobre o cancro e adaptação às repercussões na dinâmica familiar, melhorando a comunicação familiar e conferindo disponibilidade e apoio emocional.

Considerando que um modelo de IE objetiva fornecer uma base para a prática de cuidados centrados nos clientes, formularam-se os objetivos deste modelo, que demonstram os resultados que se pretendem atingir na vivência do CP pela família. Os objetivos encontram-se descritos no Quadro 1.

**Quadro 1:** Objetivos do modelo de intervenção de enfermagem – Coimbra, Portugal, 2024.

Objetivos do modelo de intervenção de enfermagem no cancro parental
Acompanhar a família que vivencia o cancro parental durante o diagnóstico, tratamento *e follow-up.*
Facilitar o processo de transição saúde-doença da figura parental com cancro.
Facilitar o processo de transição desenvolvimental do adolescente.
Facilitar a comunicação e a interação da família.
Minimizar o impacto da vivência do cancro parental na família.
Facilitar a adaptação da família às repercussões decorrentes do cancro parental.

Fonte: autores, 2024.

A finalidade do modelo é a obtenção de ganhos em saúde para os adolescentes e pais, promovendo a sua adaptação às mudanças de saúde da figura parental com cancro e na dinâmica familiar.

A estrutura do modelo compreende a organização das IEs para a família que vivencia o CP. Atendendo aos resultados da *scoping review* ^(15)^, as intervenções delineadas assentam na tipologia psicoeducacional, que integra a componente psicológica, emocional e educacional, objetivando: i) aumentar a literacia em saúde da figura parental com cancro e do adolescente sobre o CP; ii) melhorar a compreensão do adolescente e figura parental com cancro sobre a doença; iii) minimizar a angústia e medos da família relacionados com o CP; iv) promover a expressão de emoções/experiências da família; v) aumentar as competências parentais na comunicação com o adolescente; vi) orientar no processo de mudança de papéis sociais e dinâmica familiar.

Prevê-se que este modelo seja implementado por enfermeiros especialistas nas áreas da enfermagem de saúde infantil e pediátrica, e enfermagem de saúde mental e psiquiátrica.

### Metodologia da Construção do Modelo de Intervenção de Enfermagem

O modelo desenvolvido pretende responder a objetivos específicos, sendo constituído por um conjunto de objetivos, diagnósticos de enfermagem (DE), e IEs autônomas, construídas com base na Ontologia de Enfermagem.

A Ontologia de Enfermagem é um modelo de referência que representa um conjunto de conceitos do domínio disciplinar de enfermagem, bem como as relações entre estes, tendo por base a evidência científica relevante. Uma Ontologia de Enfermagem representa uma estrutura de informação com utilidade para a representação conceptual e teórica do conhecimento de enfermagem. A Ontologia de Enfermagem desenvolvida pode promover o conhecimento da enfermagem, enfatiza a necessidade de investigação, permite a evolução de sistemas que facilitem a concepção de cuidados e produzam indicadores fiáveis e de qualidade associados aos cuidados de enfermagem, reduzindo o tempo dos registos^(22)^. A Ordem dos Enfermeiros (OE)^(23)^ Portuguesa salienta ainda que a ontologia é um marco significativo para documentar a concepção dos cuidados, contribuindo para que os enfermeiros sejam cada vez mais significativos para a população, dignificando a enfermagem.

O modelo em desenvolvimento pretende representar o conhecimento em enfermagem na área do CP. O modelo configura os aspectos significativos para a satisfação das necessidades dos clientes, por meio da representação de conceitos em dados, diagnósticos, objetivos e intervenções, em uma área que vem sendo demonstrada pela evidência, resultante da pesquisa desenvolvida, representativa das necessidades efetivas dos clientes, na qual o enfermeiro pode intervir. Assim, e segundo a nomenclatura adotada pela Ontologia de Enfermagem, o modelo integra três públicos-alvo: indivíduo (figura parental com cancro); indivíduo (adolescente); e família (figura parental com cancro, segunda figura parental e adolescente).

### Conteúdos do Modelo de Intervenção de Enfermagem

Assumindo-se a filosofia dos Cuidados Centrados na Família, que preconiza planos individualizados e adaptados a cada contexto, as sessões do modelo variam de individuais (com a figura parental com cancro ou com o adolescente) a grupais, com a díade e segunda figura parental.

O modelo é constituído por quatro tópicos que visam abordar temas centrais nas diferentes fases da vivência do CP, desde o diagnóstico, tratamentos e *follow-up*, direcionados aos públicos-alvo. Os tópicos um e dois serão abordados nas sessões individuais com o progenitor doente e com o adolescente, e os tópicos três e quatro serão abordados nas sessões grupais com a família. O Quadro 2 resume os tópicos e tipo de sessões que integram o modelo de intervenção.

**Quadro 2:** Tópicos e tipo de sessão do modelo de intervenção de enfermagem – Coimbra, Portugal, 2024.

Tópicos do modelo de intervenção de enfermagem no cancro parental	Tipo de sessão
1 - *“O elefante na sala”:* vamos falar sobre o cancro parental	Individual
2 - *“Tudo muda em nós”:* como estão a vivenciar o cancro parental?	Individual
3 - *“A vida continua”:* estratégias para lidar com o cancro parental	Grupal
4 - *“Com o presente, planejamos o futuro*”: estratégias para enfrentar as consequências do cancro parental ao longo da trajetória da doença e no futuro	Grupal

Fonte: autores, 2024.

A concepção do modelo assenta nos princípios de literacia em saúde, definidos pela Organização Mundial da Saúde, pois permite aos clientes o desenvolvimento de competências cognitivas e sociais e a capacidade para aceder, compreender e utilizar a informação para promover e manter a saúde^(24)^.

Tomando como foco o processo de tomada de decisão do enfermeiro, o modelo preconiza uma abordagem centrada na família e baseada na evidência científica. Através de uma relação de parceria, é essencial que o enfermeiro demonstre conhecimentos, competências e aptidões que promovam a confiança dos adolescentes e dos pais no profissional. Assim, deve comunicar com clareza, honestidade, de forma adaptada aos clientes e transmitindo a informação de maneira compreensível, demonstrando respeito pela individualidade dos clientes. Para tal, é importante disponibilizar suporte emocional e escutar ativamente, estabelecendo um relacionamento empático e de cooperação capaz de gerar confiança, tranquilidade e vontade dos pais e adolescentes em participar e melhorar a vivência do CP.

O ambiente em que decorrerão as consultas deve ser facilitador do processo, prevendo-se que essas decorram em um espaço físico adequado, no qual se garanta a privacidade da figura parental com cancro, adolescente e família, se evitem interrupções e seja acolhedor, enquadrando-se na premissa dos cuidados atraumáticos.

O modelo baseia-se em metodologia ativa para promover as interações e relacionamentos saudáveis da díade e, simultaneamente, permitir a aquisição de conhecimentos através do desenvolvimento de um espírito crítico e reflexivo sobre a realidade, ajudando-os a ultrapassar os problemas vivenciados.

Durante as consultas de enfermagem, são fundamentais o reforço positivo e a avaliação dos resultados das intervenções implementadas, permitindo melhorar a qualidade da prestação e reavaliando as necessidades da família para as consultas subsequentes. Essas estratégias são importantes para a família valorizar a IE como um cuidado personalizado de resposta aos seus problemas.

## RESULTADOS E DISCUSSÃO

O modelo de IE para os adolescentes e pais que vivenciam o CP foi desenvolvido com recurso à Ontologia de Enfermagem, em consonância com os programas identificados na literatura e a respectiva tipologia das intervenções^(15)^, com a adequabilidade dos referenciais teóricos selecionados para os adolescentes^(16)^ e para os pais^(17)^, com as necessidades descritas pelos adolescentes^(20)^ e com a perceção dos pais sobre o impacto do cancro no desempenho do papel parental^(21)^. Segundo a Ontologia de Enfermagem, e para responder às necessidades da díade que experiencia o CP, o modelo compreende quatro etapas de concepção de cuidados que integram o processo de tomada de decisão do enfermeiro: dados a avaliar, DE, objetivos, IEs, diferenciando-se ao nível de três públicos-alvo: i) Indivíduo – figura parental com cancro; ii) Indivíduo – adolescente; iii) Família – figura parental com cancro, segunda figura parental e adolescente.

### Cliente: Indivíduo (Figura Parental com Cancro)

A figura parental com cancro experiencia repercussões da doença a vários níveis, nomeadamente no processo corporal, processo psicológico e processo de ação, o que pode comprometer o desempenho do seu papel parental, tendo implicações no comportamento interativo com os filhos. Assim, o enfermeiro deve implementar intervenções que facilitem a adaptação do progenitor com cancro a essa vivência e que promovam a interação da díade.

No que concerne ao processo corporal, é sabido que a doença oncológica e os seus tratamentos acarretam consequências físicas ao nível dos diferentes sistemas orgânicos, podendo variar de acordo com a pessoa, o tipo de cancro e o tratamento instituído. Neste sentido, na concepção deste modelo, não foram incluídos dados relativos ao processo corporal, na medida em que o que se pretende destacar é o impacto do cancro no desempenho do papel parental.

Identificaram-se os agregados conceptuais “processo psicológico - emoção” e “ação - comportamento interativo”, que resultaram nos dados, objetivos, DEs e IEs, orientados para facilitar o desempenho do papel parental no decorrer da vivência do CP.

### Cliente: Indivíduo (Adolescente)

O CP acarreta consequências no percurso de vida do adolescente, nomeadamente ao nível do processo psicológico e do processo de transição desenvolvimental. Cabe ao enfermeiro implementar intervenções que facilitem o processo adaptativo do adolescente à experiência do CP. Os agregados conceptuais identificados foram o “processo psicológico – emoção” e a “transição – desenvolvimento humano - desenvolvimento psicomotor”, que resultaram nos dados, objetivos, DEs e IEs, orientados para facilitação da vivência do evento crítico do CP.

### Cliente: Família (Figura Parental com Cancro, Segunda Figura Parental E Adolescente)

A vivência do CP implica alterações no processo familiar, em que o enfermeiro deve implementar intervenções dirigidas à figura parental com cancro, segunda figura parental e adolescente, para promover e facilitar o processo adaptativo à situação de doença.

Na Ontologia de Enfermagem, não estão previstas intervenções direcionadas às famílias que vivenciam uma doença crônica de um dos seus membros. Com a construção de um modelo de intervenção e o estudo realizado no âmbito do CP, este pode constituir-se como um impulso para a integração de um novo agregado conceptual no processo familiar “Preparação da família para se adaptar às repercussões do cancro de um membro da família”, identificando os respectivos dados, objetivos, DEs e IEs. Essa proposta sustentou-se nos resultados da *scoping review* ^(15)^ e dos estudos qualitativos^(16,17,20,21)^ que, ao evidenciarem que o CP impacta toda a família, os programas de intervenção não devem focar-se exclusivamente nos pais e/ou filhos, mas ­dirigir-se ao núcleo familiar. Assim, considerou-se pertinente que esse modelo de intervenção se dirigisse, também, à família, e não exclusivamente à figura parental com cancro e filho adolescente, possibilitando a inclusão de uma segunda figura parental.

A Figura 1 ilustra, esquematicamente, o modelo de IE que teve por referencial conceptual, teórico e semântico a Ontologia de Enfermagem, representando o conhecimento em enfermagem significativo para adolescentes e pais que vivenciam o CP.

**Figura 1:** Modelo de intervenção de enfermagem para adolescentes e pais que vivenciam o cancro parental. Coimbra, Portugal, 2024.

A sequência das sessões realizadas pelo enfermeiro será determinada de acordo com a avaliação inicial, podendo realizar-se uma primeira sessão com a figura parental com cancro, uma segunda, com o adolescente, e uma terceira, com a família. As subsequentes dependerão da avaliação realizada nas primeiras, não existindo um número pré-fixo de sessões. Podem decorrer no serviço de consulta, Hospital de Dia e/ou internamento médico/cirúrgico, e ser ministradas quer na fase de diagnóstico e/ou tratamento dos pais.

A IE requer uma abordagem colaborativa entre o adolescente e a sua família, assumindo o enfermeiro a função de facilitador das aprendizagens e veículo de transmissão de conhecimentos, ensinando, treinando e capacitando a díade. Na prática clínica, a implementação de projetos destaca-se como um contributo de boas práticas, em que o enfermeiro pode aplicar os seus conhecimentos, assumindo que, em determinado contexto, algumas soluções podem contribuir, comparativamente a outras, para a resolução de potenciais problemas^(25)^.

Atendendo aos resultados da *scoping review* ^(15)^, considerou-se pertinente que o modelo de intervenção se dirigisse, também, à família, e não exclusivamente à figura parental com cancro e filho adolescente, possibilitando a inclusão de uma segunda figura parental.

Importa salientar que a Ontologia de Enfermagem integra a taxonomia CIPE^®(26)^ para o mapeamento dos conceitos dos enunciados, sendo que os focos DE e IE, identificados nos estudos realizados com um adolescente^(16)^ e progenitor com cancro^(17)^ a vivenciar o CP, foram considerados no desenho do modelo.

O desenvolvimento do modelo fundamentou-se em intervenções de cunho psicoeducacional, porque, em conformidade com os resultados da *scoping review* ^(15)^, este tipo de intervenções não só é o mais predominante, mas parece melhor responder às necessidades da díade que se depara com o CP.

Quanto aos agregados conceptuais identificados para os diferentes clientes, pretendeu-se atender aos objetivos relativos à condição e objetivos de resultado do processo adaptativo, para minimizar os efeitos decorrentes da vivência do CP através do conhecimento, significado, capacidade e consciencialização. As IE delineadas pretendem responder às necessidades educacionais, emocionais e psicossociais dos adolescentes^(20)^ e facilitar o desempenho do papel parental do progenitor com cancro^(21)^. A partir do desenvolvimento deste modelo, resultou a proposta de integração de um novo agregado conceptual ao processo familiar: “Preparação da família para se adaptar às repercussões do cancro de um membro da família”. Este agregado, passível de integrar a Ontologia de Enfermagem, constitui-se relevante atendendo ao crescimento exponencial do CP e ao fato de a IE ser significativa para os clientes que vivenciam esse fenômeno.

As limitações deste estudo estão relacionadas à sua limitação geográfica e ao fato de que os estudos que permitiram avaliar a adequação do MTAACP para adolescentes e a relevância do MSN na abordagem aos pais foram realizados com um único participante. Destaca-se ainda o fato de o modelo ainda não ter sido implementado, considerando-se outra limitação do estudo.

Sugere-se que outras investigações sejam desenvolvidas em diferentes populações, caracterizando-as e identificando previamente as suas necessidades específicas para direcionar as intervenções aos problemas avaliados. Recomenda-se, também, que outros estudos possam explorar os níveis de intervenção de cada profissional de saúde nesse modelo.

O conhecimento do fenômeno e da sua pertinência atual deverá impulsionar a implementação desse modelo às famílias a vivenciar CP nos hospitais, onde a figura parental com cancro é acompanhada.

## CONCLUSÃO

Este estudo possibilitou desenvolver um modelo de IE para adolescentes e pais que vivenciam o CP fundamentado na evidência científica resultante de estudos realizados, que evidenciaram as necessidades dos adolescentes que vivenciam o CP e o impacto da doença do progenitor no desempenho do papel parental. O modelo sustenta-se em referenciais teóricos de enfermagem e na sua concepção, considerando-se como referencial conceptual e semântico a Ontologia de Enfermagem.

O exercício profissional dos enfermeiros insere-se em um vasto contexto de intervenção, devendo o enfermeiro assumir um papel inovador e empreendedor. Assim, como implicação para a prática clínica, a criação desse modelo de intervenção surge como uma estratégia promotora da saúde que capacita os enfermeiros para uma intervenção centrada na família. Nesse âmbito, acredita-se que os resultados desta investigação possam servir para a reformulação das políticas de saúde e de educação com base na implementação de IEs, em diferentes contextos, que minimizem as consequências do CP na díade e família que o vivencia.
